# Effects of Vitamin A Supplementation on Iron Status Indices and Iron Deficiency Anaemia: A Randomized Controlled Trial

**DOI:** 10.3390/nu6010190

**Published:** 2013-12-31

**Authors:** Hesham M. Al-Mekhlafi, Ebtesam M. Al-Zabedi, Mohamed T. Al-Maktari, Wahib M. Atroosh, Ahmed K. Al-Delaimy, Norhayati Moktar, Atiya A. Sallam, Wan Ariffin Abdullah, Rohana Jani, Johari Surin

**Affiliations:** 1 Department of Parasitology, Faculty of Medicine, University of Malaya, Kuala Lumpur 50603, Malaysia; E-Mails: wahib_atrosh@yahoo.com (W.M.A.); ahmed_soofi@yahoo.com (A.K.A.); joharisurin@um.edu.my (J.S.); 2 Department of Medical Parasitology, Faculty of Medicine, Sana’a University, Sana’a 19065, Yemen; E-Mail: mtmaktari@hotmail.com; 3 Department of Biochemistry, Faculty of Medicine, Sana’a University, Sana’a 19065, Yemen; E-Mail: somamzabedi@yahoo.com; 4 Department of Parasitology and Medical Entomology, Faculty of Medicine, Universiti Kebangsaan Malaysia, Jalan Raja Muda Abdul Aziz, Kuala Lumpur 50300, Malaysia; E-Mail: hayati@medic.ukm.my; 5 Faculty of Medicine, SEGi University College, Kota Damansara, Selangor 47810, Malaysia; E-Mail: atiya.absallam@gmail.com; 6 Department of Pediatrics, Faculty of Medicine, University of Malaya, Kuala Lumpur 50603, Malaysia; E-Mail: ariffin@ummc.edu.my; 7 Department of Applied Statistics, Faculty of Economics and Administration, University of Malaya, Kuala Lumpur 50603, Malaysia; E-Mail: rohanaj@um.edu.my

**Keywords:** clinical trial, vitamin A supplementation, iron deficiency anaemia, children, Malaysia

## Abstract

Iron deficiency anaemia (IDA) is the most common nutritional deficiency in the world including developed and developing countries. Despite intensive efforts to improve the quality of life of rural and aboriginal communities in Malaysia, anaemia and IDA are still major public health problems in these communities particularly among children. A randomized, double-blind, placebo-controlled trial was conducted on 250 Orang Asli (aboriginal) schoolchildren in Malaysia to investigate the effects of a single high-dose of vitamin A supplementation (200,000 IU) on iron status indices, anaemia and IDA status. The effect of the supplement was assessed after 3 months of receiving the supplements; after a complete 3-day deworming course of 400 mg/day of albendazole tablets. The prevalence of anaemia was found to be high: 48.5% (95% CI = 42.3, 54.8). Moreover, 34% (95% CI = 28.3, 40.2) of the children had IDA, which accounted for 70.1% of the anaemic cases. The findings showed that the reduction in serum ferritin level and the increments in haemoglobin, serum iron and transferrin saturation were found to be significant among children allocated to the vitamin A group compared to those allocated to the placebo group (*p* < 0.01). Moreover, a significant reduction in the prevalence of IDA by almost 22% than prevalence at baseline was reported among children in the vitamin A group compared with only 2.3% reduction among children in the placebo group. In conclusion, vitamin A supplementation showed a significant impact on iron status indices and IDA among Orang Asli children. Hence, providing vitamin A supplementation and imparting the knowledge related to nutritious food should be considered in the efforts to improve the nutritional and health status of these children as a part of efforts to improve the quality of life in rural and aboriginal communities.

## 1. Introduction

Iron deficiency anaemia (IDA) is reported to be the most common nutritional deficiency in the world [1,2]. It affects almost one-third of the world’s population and it is common in young children [3]. Prevalence of IDA has been reported as high as 50% among East-Asian children of school age [4], and 60% among children less than 5 years [5]. Furthermore, the prevalence of IDA in children living at the periphery of large cities in the USA was found to be similar to that observed in developing countries [6]. Overall, preschool and school-age children, adolescent females and pregnant women are the groups at risk to develop IDA [1,7]. Iron deficiency anaemia in children is associated with decreased physical capacity and growth, impaired immune system, and reduced cognitive functions and learning capacities [8,9].

Iron deficiency anaemia, vitamin A deficiency (VAD) and helminthes infections, mainly soil-transmitted helminths (STH) coexist among low-income populations. A possible association between vitamin A and iron has been suggested previously and serious attention has been given to this relationship [10,11,12,13]. For logistic reasons, the World Health Organization (WHO, Geneva, Switzerland) has recommended that vitamin A capsules and anthelmintic tablets be delivered together [14].

In Malaysia, despite intensive efforts to improve the quality of life among rural and aboriginal communities, several studies showed that anaemia, IDA, VAD and STH were highly prevalent, particularly among children [15,16,17,18]. A significant association between IDA and low serum retinol was reported [13]. However, this study was based on a single point data analysis (cross-sectional), and it therefore did not explain causality. Within this context, the aim of the present study was to investigate the effects of vitamin A supplementation on iron status indices and IDA status among Orang Asli primary schoolchildren in rural Malaysia.

## 2. Methods

### 2.1. Study Design

This study was a randomized, double-blind, placebo-controlled trial (Trial Registration: clinicaltrials.gov; identifier: NCT00936091; National Institutes of Health, Bethesda, MD, USA). After baseline screening for the eligibility of the children, 250 eligible children were assigned randomly into two groups (125 children per group) to receive either vitamin A supplement or its identical placebo. At the school, neither the person administering the supplements, nor the child receiving the capsule, was aware of the intervention. Blood samples were coded and the person who processed and analyzed the samples was not aware which treatment group any sample corresponded to.

### 2.2. Study Area

**Figure 1:**
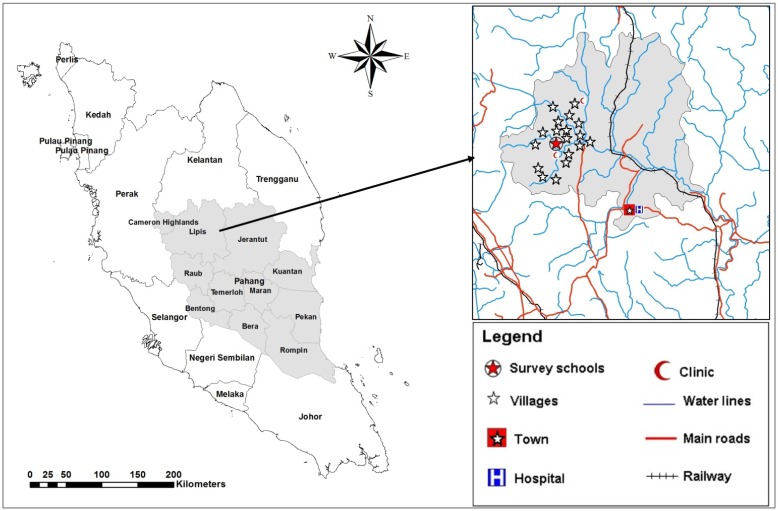
A geographic map showing the study area (location of school and villages in Lipis district).

This study was conducted in the Lipis district of Pahang state, Malaysia; 200 km northeast of Kuala Lumpur, Malaysia. The study area consisted of 18 villages located in a valley region and considered a remote area (Figure 1). There is a clinic at the area for health services equipped with an ambulance to send critical cases to the nearest hospital at Kuala Lipis, the main town of Lipis district (50 km). Aboriginal people live in houses made of wood or bamboo. However, most of the houses have electricity during the night time only and a supply of piped water as the main source for drinking water, while water for domestic needs (bathing, washing clothes and utensils, and feeding animals) is collected from the rivers located adjacent to the villages. Most of the residents at this area work as farmers, labourers, rubber tappers and some do other jobs such as selling forest products. Primary schoolchildren of Sekolah Kebangsaan Betau (the Betau National School, Pahang, Malaysia), a primary school for aboriginal children, were selected for this study.

### 2.3. Study Population

**Figure 2:**
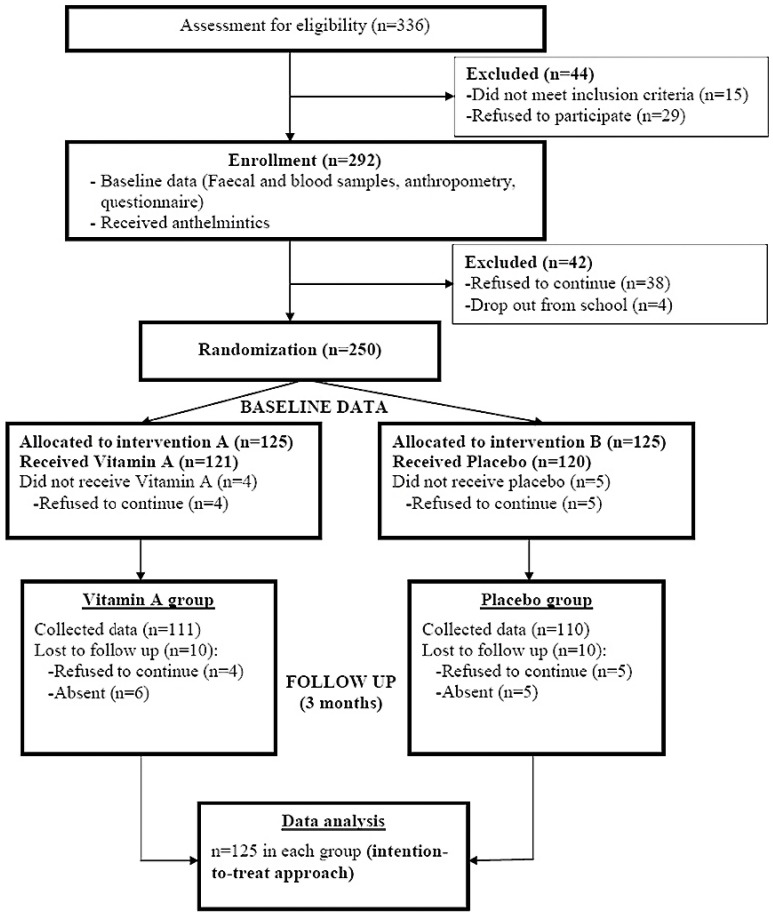
Flow chart of data collection and follow up.

This study was conducted among Orang Asli schoolchildren. Orang Asli are the indigenous minority peoples of Peninsular Malaysia and the name, Orang Asli, is a Malay term translated as “original or first people”. The total number of Orang Asli represents 0.7% of the country’s total population. A sample size of 214 children, 107 per intervention arm, was estimated to give the study at least 80% power at 5% significance to detect a 10% or more difference in the prevalence and intensity of parasitic infections between the vitamin A-supplemented group and the placebo group. The school had an enrolment of 502 pupils in grades one through six. There were 405 pupils in the target age range of 7–12 years. Of these, 69 were absent at the time of enrolment, 29 refused to participate, and 15 were excluded because they had infections with fever at the time of enrolment. Finally, faecal and blood samples were collected from 292 eligible children and used for baseline assessment. They received a 3-day course of anthelmintics and subsequently 250 of them agreed to participate in the intervention part of this study. This calculation includes 20% more subjects to avoid those that may become lost to follow-up). Descriptions of the trial profile, data collection and follow-up were illustrated according to the CONSORT guideline and shown in Figure 2.

### 2.4. Ethical Consideration

This study was conducted according to the guidelines laid down in the Declaration of Helsinki [19] and the protocol was approved by the Medical Ethics Committee of the University of Malaya Medical Centre, University of Malaya, Kuala Lumpur, Malaysia. Before the commencement of the present study, community meetings were held with the headmaster, heads of the villages, parents and their school-age children in order to give a clear explanation of the objectives, procedures and the involvement of the children in this study. During the meeting, they were also informed that their participation was totally voluntarily and they could decide to withdraw from the study at any time without assigning any reason whatsoever. Informed consent was obtained from the participants and their guardians. Thus, verbal informed consents were taken from parents or guardians, on behalf of their children. Verbal consents were recorded and these procedures were approved by the Medical Ethics Committee of the University of Malaya Medical Centre. All the infected children were treated with a 3-day course of 400 mg albendazole tablets as a part of the procedures of this study.

### 2.5. Interventions

Vitamin A supplements and placebos were provided by Tablets (India) Ltd. (Chennai, India) and were gelatinous and reddish opaque capsules containing 200,000 IU vitamin A in 200 μL peanut oil with 10 μg vitamin E as a preservative or identical capsules containing the 200 μL peanut oil with 10 μg vitamin E only. The capsules were provided in identical, dark brown and well-sealed glass bottles containing 50 capsules each. The capsules were encoded (A and B) and the code was kept confidential by personnel who were not involved in the study until the study ended and the code was broken. Each child received the capsule of the relevant group under a direct observed therapy. The dose was followed by two pieces of freshly fried banana (locally known as Pisang Goreng) which is rich in oil that provides a perfect medium for vitamin A absorption and ensures maximum utilization of the capsule’s contents. Fortunately, these fried bananas are a favourite snack in Malaysia and were readily accepted by the recruited children.

Albendazole tablets (GlaxoSmithKline, London, UK) were also used in this study as the anthelmintic treatment and the regime used was a 3-day course of 400 mg/day. Each white chewable tablet contains 400 mg albendazole as the active ingredient. Tablets were provided in white, well-sealed plastic bottles containing 100 tablets each. Each child chewed the tablet before swallowing it with some water, while being observed by a researcher, medical officer, and a teacher (Directly Observed Therapy). The efficacy of the treatment was assessed after 12–14 days, and children still infected were treated again with a single dose of albendazole 400 mg.

### 2.6. Parasitology

Fresh faecal samples were collected into wide-mouth screw-cap 100 mL clean containers. The samples were examined by the Kato–Katz and Harada Mori techniques for the presence of STH, *Ascaris lumbricoides*, *Trichuris* *trichiura* and hookworm eggs [20,21]. Egg counts, as a measure of worm burden, were also carried out using this technique and the results were recorded as eggs per gram of stool (epg). The intensity of infection was graded as heavy, moderate or light according to criteria proposed by the WHO [22]. Therefore, scores for the intensity of infections were given to each STH species (light = 1; mild = 2; and heavy = 3) and infections with worm score ≥ 5 were included in the analysis.

### 2.7. Haematological and Biochemical Analysis

About 3–5 mL of venous blood was collected from each subject into a plain tube for biochemical analysis. Haemoglobin (Hb) concentration was measured directly after blood withdrawal using portable HemoCue haemoglobinometer (HemoCue Hb 201 DM, company, Angelhom, Sweden). Hb concentration was recorded as g/dL. Children with Hb levels lower than 12 g/dL were considered as anaemic [1,23]. The blood was left at room temperature for clot formation then the tubes were centrifuged at 3,000 rpm for 10 min to obtain the serum that was stored at −20 °C till further analysis.

Serum ferritin (SF) levels were analyzed by means of the ADVIA Centaur Analyzer (Siemens Medical Solutions Diagnostics, Tarrytown, NY, USA), and children with concentrations of less than 10 μg/L were considered to have deficient iron stores. Meanwhile, serum iron (SI) and TIBC (total iron-binding capacity) were determined colourimetrically using Cary 50 analyzer (Varian UV-VIS-NIR, Varian, Inc., Palo Alto, CA, USA), and then the percentage transferrin saturation (TS) was calculated from the ratio of SI concentration to TIBC [15]. For quality control, 20% of the samples were randomly selected and examined in duplicate. Children were identified to have IDA if they were anaemic and had low SF (less than 10 μg/L) and/or low SI (less than 10.6 μmol/L), high TIBC (more than 75 μmol/L) and low TS (less than 16%) [13,23,24].

Serum retinol (SR) levels were determined using a Reverse Phase High Performance Liquid Chromatography (HPLC) (LC-10AD, Shimadzu, Kyoto, Japan) as described earlier [25,26] with suitable modifications. Serum retinol level was recorded as μmol/L. In order to minimize the effects of inflammation on the iron status indices and retinol analysis, C-reactive protein (CRP) level was measured and children with evidence of inflammation were excluded from the study. In addition, a small cut-off point was used with SF; when ferritin levels are low (<10 μg/L) there is no doubt that serum iron status is deficient [27].

### 2.8. Nutrient Intakes

Dietary intakes of the children were assessed by using 24-h dietary recall and food frequency questionnaire. Subjects (or parents for those who could not recall their 24-h diet) were asked to recall all the foods and beverages consumed the day before. Then, calories and nutrient intakes were calculated using a nutrient analysis computer programme “Nutritionist Pro” (Axxya Systems LLC, Stafford, TX, USA) with consideration for the Malaysian food composition tables compiled by [28]. The Malaysian recommended daily intake (RDA) for calories, protein, vitamin A and iron was used to compare the daily intake and to evaluate the nutrient adequacy of these children [29]. The food frequency questionnaire (FFQ) was also used to characterize the habitual intakes of individual children for calories, protein, iron and vitamin A, in both its forms: preformed vitamin A (retinol) and provitamin A (carotene). This FFQ covered 60 foods containing the mentioned elements and commonly found in this community. Local Malay names of these foods were identified during the preliminary surveys to explore the area and the school.

### 2.9. Statistical Analysis

Statistical analysis of data was done using Statistical Package for Social Sciences for Windows SPSS (version 13, SPSS Inc., Chicago, IL, USA). The distribution of iron status indices and retinol was examined for normality using the Shapiro-Wilk test. For descriptive analysis, proportion with 95% confidence interval (CI) was used to present the prevalence of categorical variables. The distribution of quantitative variables was examined for normality using the Kolmogorov-Smirnov *Z* test before analysis, and mean and standard deviation (SD) were used to present the normally distributed variables, whereas variables that were not normally distributed were presented as median and interquartile range (IQR). To compare the level of iron status indices between groups, the independent *t*-test or Mann-Whitney *U* test were used where applicable. Changes in these parameters between the baseline and the follow-up assessment (3 months) were presented as mean and 95% CI and examined by paired samples *t*-test or Wilcoxon test, whichever suitable. Moreover, the chi-square test was used to examine the significance of the differences in the frequency distribution of categorical variables. Correlations between the levels of SR at baseline and iron status indices levels were examined by Pearson’s and Spearman correlation coefficients.

The intervention of this study was vitamin A supplements, and low serum retinol showed a significant association with IDA in combination with other factors including age, gender and maternal education [13]. Thus, changes in the levels of Hb and iron status indices were compared according to these factors. Moreover, in order to avoid bias and violation of power of study due to missing data after randomization, intention-to-treat analysis (ITT) was used to analyze the effects of intervention on the dependent variables [30,31]. Therefore, all randomized children were analyzed in the group they originally assigned. All tests were considered significant at *p* < 0.05.

## 3. Results

### 3.1. General Baseline Characteristics of Subjects Involved in the Trial

A total of 250 eligible schoolchildren (126 males, 124 females) aged 7–12 years with a median age of 10 years (IQR 8–11) agreed to participate in this intervention study. The baseline characteristics of the schoolchildren involved in this intervention study are shown in Table 1. There were no significant differences in the variables and characteristics between the two groups and this indicated that the randomization process distributed all variables and possible confounders equally between the two groups.

### 3.2. Iron Status Indices, Anaemia and IDA (Iron Deficiency Anaemia) Status

The total percentage of children with low SF, SI and TS concentrations was 26.1%, 49.4% and 52.7%, respectively and high TIBC was reported in 63.9% of the children.

**Table 1:** Baseline characteristics of the intervention groups *. TIBC, total iron binding capacity; IDA, iron deficiency anaemia.

Characteristics	Vitamin A	Placebo
*N*	125	125
Male/Female	63/62	63/62
Age (years) ^a^	10 (8, 11)	10 (9, 11)
Haemoglobin (g/dL) ^a^	12.1 (10.3, 12.2)	12.0 (10.2, 12.2)
Serum ferritin (μg/L) ^b^	15.5 ± 6.8	15.7 ± 6.7
Serum iron (μmol/L) ^a^	10.8(9.9, 15.3)	11.2(9.7, 15.7)
TIBC (μmol/L) ^b^	72.8 ± 8.5	72.4 ± 10.7
Transferrin Saturation ^b^	17.7 ± 6.9	18.3 ± 8.0
Anaemia ^c^	59 (48.8)	58 (48.3)
IDA ^c^	43 (35.5)	39 (32.5)
Serum retinol (μmol/L) ^a^	1.12 (0.69, 1.47)	1.14 (0.69, 1.40)
Ascariasis (%)	87 (69.6)	82 (65.6)
Trichuriasis (%)	120 (96.0)	122 (97.6)
Hookworm infections (%)	18 (14.4)	13 (10.4)
Giardiasis (%)	18 (14.4)	27 (21.6)

* All differences were non-significant; *N*: Represents the number of subjects; ^a^ Median (IQR), *p*-values for the differences between vitamin A and placebo groups (Mann-Whitney *U* test); ^b^ Mean ± SD, *p*-values for the differences between vitamin A and placebo groups (independent *t*-test); ^c^ Number (%) *p*-values for the differences between vitamin A and placebo groups (Chi-square test).

Overall, 117 (48.5%; 95% CI = 42.3, 54.8) children were anaemic (Hb < 12.0 g/dL). The prevalence of IDA was 34% (95% CI = 28.3, 40.2), which accounted for 70.1% of the anaemic cases among the subjects. The prevalence of anaemia was significantly higher among children aged ≤10 years than children aged >10 years (χ^2^ = 3.744; *p* = 0.050). There was no significant difference in the prevalence of anaemia between males and females (χ^2^ = 2.191; *p* = 0.139).

Correlations between the levels of SR and iron status indices levels at baseline are shown in Table 2. Overall, there were significant positive correlations between changes of SR and Hb, SF and TS. However, the correlation of SR with SF was weak (*p* < 0.05). When the correlation was compared according to age groups and gender, the correlation between SR and Hb remained significant among females and children aged ≤10 years while the correlation between SR and Hb among males and children aged >10 years turned out not to be significant (*p* > 0.05). Similarly, SR correlated significantly with SF among females only. On the other hand, the correlation between SR and SF was not significant.

**Table 2:** Correlation between serum retinol and iron status indices at baseline according to age and gender. SR, serum retinol; Hb, haemoglobin; SF, serum ferritin; TS, transferrin saturation.

Gender/Age Groups	*N*	SR + Hb	SR + SF	SR + TS
*All*	241	0.276 *	0.195 *	0.331 **
*Age Group (Years)*				
≤10 years	167	0.224 *	0.170	0.266 *
>10 years	74	0.202	0.166	0.473 **
*Gender*				
Males	120	0.186	0.111	0.284 **
Females	121	0.252 *	0.282 **	0.305 **

* Correlation was significant at the 0.05 level; ** Correlation was significant at the 0.01 level.

### 3.3. Effects of Vitamin A Supplementation on Iron Status Indices, Anaemia and IDA

The mean changes in the levels of Hb and iron status indices are shown in Table 3. The changes of all indices except TIBC were found to be significantly higher among children allocated to vitamin A group than those allocated to placebo group (*p* < 0.01).

**Table 3:** Mean changes in Hb and iron status indices from baseline 3 months after intervention in the vitamin A and placebo groups ^a^.

Variable	Vitamin A	Placebo
Haemoglobin (g/dL)	0.51 (0.40, 0.56) ^b,1^	0.21 (0.15, 0.28)
Serum ferritin (μg/L)	−1.50 (−2.68, −0.33) ^b,2^	0.90 (0.20, 1.61)
Serum iron (μmol/L)	1.60 (1.16, 2.0) ^b,2^	0.49 (0.02, 0.98)
TIBC (μmol/L)	−4.30 (−6.18, −2.44)	−1.53 (−3.64, 0.58)
Transferrin saturation	4.38 (3.15, 5.62) ^b,2^	1.34 (0.95, 2.7)

^a^ All values are mean (95% CI); ^b^ Significant difference from placebo group (independent *t*-test): (^1^* p* < 0.001, ^2^* p* < 0.01).

There was a reduction in the SF levels at three months by 1.50 μg/L of baseline levels in the vitamin A group and this change was significantly (*p* < 0.01) higher than that in the placebo group. Similarly, the mean changes (reduction) in TIBC was higher in the vitamin A group than the placebo group but this difference was not statistically significant (*p* > 0.05). By comparing these changes among the serum retinol status categories, there were no significant differences in changes according to serum retinol status at baseline.

Although there were reductions in the prevalence of anaemia and IDA at three months compared to the baseline prevalence, this reduction was only significant for the prevalence of IDA among children in the vitamin A group (Table 4). These children showed significant reduction in the prevalence of IDA by 22.4% than prevalence at baseline compared with only 5.6% reduction among children in the placebo group. Similarly, vitamin A-supplemented children showed a reduction in the prevalence of anaemia by 20.0% over the prevalence at baseline compared to 14.4% among those in the placebo group.

**Table 4:** Effects of vitamin A supplementation on prevalence of anaemia and IDA after 3 months.

Group	Prevalence of anaemia (%)		Prevalence of IDA (%)	
	Baseline	After 3 months	Baseline	After 3 months
Vitamin A	59 (47.2)	34 (27.2)	43 (34.4)	15 (12.0)
Placebo	58 (46.4)	40 (32.0)	39 (31.2)	32 (25.6)
*P*	0.947	0.378	0.619	0.005

Values are number of anaemic cases (%); *P*: *p*-value for the difference between the two groups (Chi-square test).

The analysis of Hb and iron status indices changes over a period of 3 months were compared according to age groups, gender, mothers’ educational level and serum retinol status at baseline, and the results are presented in Table 5 and Table 6. Table 5 shows that the significant impact of vitamin A supplementation was mainly amongst younger children (≤10 years), whereas the changes in Hb and SF among children aged >10 years were not significantly different than control children. Mothers’ education showed an important influence in that those with non-educated mothers could not benefit significantly from the vitamin A supplementation. As is consistent with previous results, those with satisfactory baseline vitamin A status showed significant changes in SF. Moreover, Table 6 shows a similar scenario for the effects of these variables on the changes in SI, TIBC and TS as responded to the interventions (vitamin A and placebo).

**Table 5:** Mean changes in Hb and SF among schoolchildren in the vitamin A and placebo groups according to selected variables.

Variables	Haemoglobin (g/dL)		Serum ferritin (μg/L)	
	Vitamin A	Placebo	Vitamin A	Placebo
*Age Groups*				
≤10 years	0.56 (0.4–0.7) ^a,b^^,^^1^	0.18 (0.1–0.3)	−2.1 (−3.7–−0.5) ^b^^,^^2^	1.7 (0.5–2.8) ^a^
>10 years	0.48 (0.3–0.6)	0.25 (0.2–0.5)	−0.8 (−3.0–1.3)	−0.9 (−3.7–1.3)
*Gender*				
Males	0.47 (0.4–0.6) ^b^^,^^3^	0.25 (0.1–0.4)	−2.9 (−4.8–−1.1) ^b^^,^^1^	1.4 (−0.4–3.1)
Females	0.54 (0.4–0.7) ^b^^,^^1^	0.19 (0.1–0.3)	−0.59 (−2.4–1.2)	0.77 (−0.7–2.2)
*Educational Levels of Mothers*				
≥6 years formal education	0.39 (0.2–0.5) ^b^^,^^3^	0.23 (0.1–0.3)	−4.3 (−6.9–−1.6) ^b^^,^^1^	1.5 (0.4–2.7)
No formal education	0.53 (0.4–0.6) ^b^^,^^1^	0.15 (–0.1–0.3)	−1.2 (−2.6–0.3)	−0.7 (−4.1–2.6)
*Serum Retinol Status*				
Low (<10 μg/L)	0.53 (0.1–0.6) ^b^^,^^1^	0.13 (0.01–0.3)	−0.1 (−2.2–2.2)	0.9 (−1.1–2.9)
Normal (≥10 μg/L)	0.50 (0.4–0.6) ^b^^,^^2^	0.24 (0.2–0.3)	−2.5 (−4.1–−0.9) ^b^^,^^2^	1.1 (−0.2–2.5)

Values are mean (95% CI); ^a^ Significant difference between the variable groups: independent *t*-test (*p* < 0.05); ^b^ Significant difference between the vitamin and placebo groups: independent *t*-test (^1^ *p* < 0.001, ^2^ *p* < 0.01, ^3^ *p* < 0.05).

**Table 6:** Mean changes in SI, TIBC and TS among schoolchildren in the vitamin A and placebo groups according to selected variables.

Variable	Serum Iron (μmol/L)		TIBC (μmol/L)		Transferrin Saturation	
	Vitamin A	Placebo	Vitamin A	Placebo	Vitamin A	Placebo
*Age Groups*						
≤10 years	1.4 (0.9–1.9) ^b,1^	0.3 (−0.4–0.9)	−5.9 (−7.2–−2.4) ^b,1^	−1.1 (−3.5–1.4)	4.4 (2.8–5.9) ^b,2^	1.7 (−0.4–2.5)
>10 years	2.1 (1.2–3.1) ^b,3^	1.1 (0.1–2.1)	−3.9 (−8.1–0.2)	−3.5 (−6.1–4.2)	5.4 (2.5–8.3) ^b,3^	2.7 (−1.4–4.6)
*Gender*						
Males	1.8 (1.1–2.4) ^b,2^	0.5 (−0.3– 0.9)	−5.0 (−7.9–−2.1) ^b,1^	1.1 (−1.9–4.1)	5.0 (3.0–7.1) ^b,1^	0.2 (−1.7–2.0)
Females	1.5 (0.9–2.1)	0.8 (0.1–1.4)	−4.2 (−4.1–−1.2)	−4.1(−7.8–−0.4) ^a^	4.3 (2.5–6.1)	2.5 (0.5–4.5) ^a^
*Educational Levels of Mothers*						
≥6 years formal education	1.6 (0.3–2.8) ^b,2^	0.4 (−0.2–1.0)	−4.1 (−9.2–0.9) ^b,1^	−0.5 (–3.2–2.1)	4.7 (0.4–8.9) ^b,2^	1.5 (−0.5–2.6)
No formal education	1.6 (1.1–2.1) ^b,3^	0.6 (−0.9–2.0)	−4.7 (−6.9–−2.4)	−5.3 (–7.4–0.5) ^a^	4.7 (3.3–6.1)	2.3 (−0.9–5.5)
*Serum Retinol Status*						
Low (<10 μg/L)	1.6 (1.0–2.1)	0.8 (0.2–1.7)	−4.4 (–8.3–−0.5) ^b,3^	−1.9 (−6.3–2.4)	3.9 (1.6–6.2) ^b,3^	1.7 (−0.4–3.7)
Normal (≥10 μg/L)	1.6 (1.1–2.2) ^b,2^	0.3 (−0.4–0.7)	−4.7 (−7.1–−2.2) ^b,2^	−1.4 (−4.3–1.5)	5.1 (3.3–6.7) ^b,2^	1.2 (−0.6–2.9)

Values are mean (95% CI); ^a^ Significant difference between the variable groups: independent *t*-test (*p* < 0.05); ^b^ Significant difference between the vitamin and placebo groups: independent *t*-test (^1^* p* < 0.001, ^2^* p* < 0.01, ^3^* p* < 0.05).

### 3.4. Nutrient Intakes

The mean daily intakes of iron and vitamin A were analyzed according to age, gender and treatment group. Overall, the daily nutrient intakes of Orang Asli schoolchildren were unsatisfactory; there were deficits in the intakes of energy, iron and vitamin A as compared to the RDA. Generally, the nutrient intakes were increased by age without significant differences. When these intakes were compared according to gender, females appeared to have better intakes than males, with the exception of iron intake. Females’ iron intakes were significantly lower than males (6.7 ± 3.2 mg compared to 8.1 ± 2.7 mg). On the other hand, the energy intakes were significantly higher among females than males (1256.2 ± 295.1 kcal compared to 1032.3 ± 303.5 kcal). Moreover, the overall vitamin A intake was 525.3 ± 321.3 μg RE (541.2 ± 328.3 for males and 533.7 ± 316.5 for females).

## 4. Discussion

Although anaemia has been recognized as a public health problem for many years, little progress has been reported in the control of anaemia; the global prevalence remains unacceptably high. It is estimated that most children and pregnant women in developing countries and 40% in developed countries are iron deficient [1]. Findings of the present study revealed high prevalence of anaemia and IDA. Almost half (48.5%) of the children were anaemic and the prevalence of IDA was 34% and accounted for 70.1% of the anaemic cases. These findings are in agreement with previous studies conducted among rural communities in different states of Malaysia [13,17,23]. These findings vividly revealed the magnitude of iron deficiency among rural and aboriginal populations. Poverty is dominant in these communities and the high prevalence of anaemia and IDA could be attributed to the poor availability of food stuffs and health care. Our findings are also in accordance with the WHO global estimates that up to 48% of school-age children in developing countries have IDA [1].

In the present study, we also present data that shows the effects of vitamin A supplementation of iron status indices and prevalence of IDA among Orang Asli schoolchildren in Malaysia. We found a significant association between IDA and vitamin A deficiency (VAD) represented by low serum retinol. A possible association between iron and vitamin A was suggested in many previous studies and serious attention has been given to this relationship [10,11,12,13,32]. Although the levels of Hb, SI and TS were significantly higher in both groups when compared with baseline assessment, our findings clearly indicated that the changes in Hb and other iron status indices after three months were significantly higher in the vitamin A-supplemented children compared to placebo-supplemented children (*p* < 0.01). In accordance to the general effect, levels of SF and TIBC after three months were significantly lower than baseline measurements among children in the vitamin A group compared with children in the placebo group. These findings were in agreement with previous studies in other countries [32,33,34,35].

Previous studies among Indonesian children support our findings and reported significant improvements in Hb concentration among vitamin A-supplemented children compared to control children [36,37]. The increments were much higher when vitamin A was distributed together with albendazole tablets in children infected with *A*. *lumbricoides* and/or *T*. *trichiura* [37]. In a controlled, clinical trial involving Indonesian preschool children with clinical and sub-clinical VAD, vitamin A supplementation (200,000 IU) was associated with a significant increase of 2.1 g/dL haemoglobin among those children who were anaemic at enrolment [33]. Similarly, a study among pregnant women in West Java, Indonesia showed that the increase in Hb was more than 50% greater when vitamin A was supplied with iron compared to individual element and this combination was sufficient to eliminate anaemia in 97% of anaemic women at baseline [34]. In comparison, the mean increments in Hb concentration among vitamin A-supplemented children reported by the present study were much lower than that reported by Mwanri *et al.* [32] for the same period of time. This could be due to the scope of their study which is confined to anaemic children who show more improvements than non-anaemic children or the difference in the daily intake of iron. However, we found that when the mean changes of iron status indices were adjusted by anaemia status at baseline, the mean changes of Hb, SF and TS were significantly higher among anaemic children compared to non-anaemic children (*p* < 0.01).

In contrast to these findings, a previous study found no effect for weekly vitamin A supplementation on Hb concentration among schoolchildren in rural and urban East Java, Indonesia [38]. This could be due to the short duration and low vitamin A dose (10,000 IU) used. Moreover, the prevalence of anaemia was almost half of the prevalence reported among Orang Asli children by the present study. Moreover, the benefits of vitamin A supplements appear to be more pronounced among anaemic children [32]. In the same vein, iron and vitamin A daily intake among the studied population was found to be low. Optimal benefits of the deworming and vitamin A supplementation would be achieved with improvement of the daily intake of these elements and this could be achieved by promoting health education pertaining to the nutritious and balanced diet together with appropriate interventions for poverty alleviation.

Interestingly, the present study reported a reduction rather than increase in SF level as the level after three months of receiving vitamin A supplements was lower compared to baseline assessment (14.34 μg/L compared to 15.5 μg/L), whereas children who received placebo had almost the same level at baseline and after three months. The expected effect of vitamin A in reducing the effects of infections and improving the haematopoiesis could explain this observation. Meanwhile, there was a greater reduction in TIBC levels in the vitamin A group than placebo group. A marked elevation in SF during the acute and chronic phases of inflammation was reported even in the presence of marked iron deficiency [39]. It is possible that that the high prevalence of iron deficiency reported by our study was masked by higher infection rates at baseline. Therefore, a decline in infection rate as a result of vitamin A supplementation may contribute to the decrease in SF levels.

A study by Muslimatun [35] found significant increments in Hb concentration among Indonesian pregnant women who received weekly vitamin A and iron supplementations compared to women who received weekly iron or daily iron only. The authors also indicated that there was a sharp decline in SF levels among women who received vitamin A and iron, suggesting to the investigators that vitamin A supplementations improve the utilization of iron from body stores for haematopoiesis. Similarly, a high dose of vitamin A significantly increased the mean haemoglobin level by 0.7 g/dL and reduced the prevalence of anaemia from 54% to 38% over a period of 5–10 months among Moroccan children deficient in vitamin A and iron [40]. It was concluded that this effect was independent of iron status and vitamin A supplementation does not enhance iron absorption and increases body iron stores but it increases the production of erythropoietin which mobilizes iron from existing stores to support increased erythropoiesis [40]. Hence, providing vitamin A and iron supplementations together with health education about nutritious food should be also considered in communities with poor dietary intake of iron and vitamin A.

By comparing the prevalence of anaemia and IDA at baseline and after three months, it is clear that there was a reduction in the prevalence of anaemia and IDA by three months. However, this reduction was only significant in the prevalence of IDA among vitamin A-supplementedchildren (*p* < 0.01), and this supports the significant impact of vitamin A on Hb concentration and iron status indices. Among Indonesian preschool children, a significant decrease in the proportion of anaemia among recipients of vitamin A supplements (200,000 IU) from 25.7% to 15.3% was reported after two months, whereas the proportion did not show a significant change after supplementation in non-recipients [36]. Moreover, combined vitamin A and iron supplementation were shown to reduce anaemia more effectively [41].

## 5. Conclusions

Our findings provide a population-based picture of iron status indices among Orang Asli children living in underprivileged rural areas. The prevalence of anaemia and IDA among these children was found to be high, and these findings thus support the urgent need to identify integrated and effective control measures to manage significantly these problems in these communities. Our findings showed a significant impact for vitamin A supplementation in improving iron status indices and reducing IDA. Hence, vitamin A supplementation and health education on nutrition should be included in public health strategies for the control and prevention of anaemia and IDA in rural areas.
